# Ectopic and molar pregnancies in Brazil: A secondary analysis of the WHO multi‐country survey on abortion

**DOI:** 10.1002/ijgo.70142

**Published:** 2025-04-07

**Authors:** Luís Henrique Leão, Camila Ayume Amano Cavalari, Luiz Francisco Baccaro

**Affiliations:** ^1^ Departamento de Tocoginecologia, Faculdade de Ciências Médicas Universidade Estadual de Campinas (UNICAMP) São Paulo Brazil

**Keywords:** ectopic pregnancy, hydatidiform mole, pregnancy complications, spontaneous abortion

Ectopic pregnancy (EP) and molar pregnancy (MP) are serious complications in early pregnancy that can lead to severe maternal health risks and even death if not promptly diagnosed and managed. Early detection and intervention are crucial to reducing adverse outcomes.^1^ Data on EP and MP in Brazil are scarce. This study aims to describe the prevalence, severity, and management of EP and MP among women treated for early pregnancy loss in Brazil.

Our study is a secondary analysis of the WHO Multi‐Country Survey on Abortion (WHOMCS‐A), specifically examining women diagnosed with EP or MP in Brazil. The WHOMCS‐A was a large, cross‐sectional study conducted across 280 healthcare facilities in 17 countries in Africa and Latin America and the Caribbean (LAC), focusing on early pregnancy loss (including abortion, miscarriage, EP and MP). The study protocol and main findings, including the recruitment process and data collection methods, have been previously published.^2^, ^3^, ^4^ The study protocol received ethical approval from the WHO Research Ethics Review Committee (protocol: 0002699), Brazil's National Institutional Review Board, and the respective local Research Ethics Committees (CAAE: 78745317.1.0000.5404).

We classified women into four groups based on complication severity: severe maternal outcome (SMO), potentially life‐threatening conditions (PLTCs), moderate, and mild complications. Management approaches were categorized as surgical or clinical. Surgical treatments included uterine evacuation (dilation and curettage, manual aspiration), laparotomy, and laparoscopy. Clinical treatments comprised medical therapies (methotrexate), uterotonics (misoprostol, oxytocin), intravenous (IV) fluids, vasopressors, antibiotics, procoagulant agents, blood transfusions, intensive care unit admissions, and extended hospital stays. Statistical analyses included chi‐square, Fisher exact test, and Mann–Whitney test, using SAS version 9.2.

The total number of women included in the WHOMCS‐A database in Brazil was 1868. The total number of women with EP and MP across the database was 9.9% (185/1868) whereby EP accounted for 7.4% (138/1868) and MP for 2.5% (47/1868). Women with EP had a higher mean age (29.7 ± 5.8 years) than those with MP (26.9 ± 8.1 years, *P* < 0.01). Most of the women with EP presented with a lower gestational age (91.7% with <12 weeks' gestation) compared with women with MP (55.3% with <12 weeks' gestation). EP cases had higher rates of PLTCs (8.7% vs. 4.3%) and moderate complications (37.7% vs. 14.9%) than MP cases, while MP had more mild complications (78.7% vs. 52.9%) (Figure 1).

**FIGURE 1:**
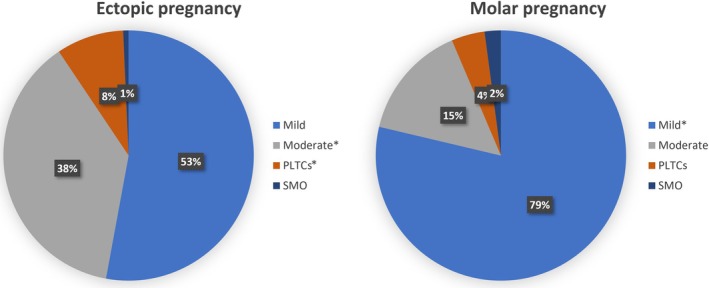
Severity of complications in ectopic pregnancy (EP; *n* = 138) and molar pregnancy (MP; *n* = 47). Chi‐square test for comparison between EP and MP. *P < 0.05. PLTCs, potentially life‐threatening complications; SMO, severe maternal outcome.

At admission, most EP patients presented abdominal pain (74.6%), vaginal bleeding (63%), and peritoneal irritation (34.1%). MP cases commonly presented with vaginal bleeding (76.6%), an enlarged uterus (53.2%), and vomiting (12.8%). Compared with MP cases, EP cases more frequently required laparotomy (82.6% vs. 0%), prolonged hospitalization (33.1% vs. 12.8%), IV fluids (94.2% vs. 72.3%), and antibiotics (60.6% vs. 42.5%). MP cases were primarily managed by uterine evacuation (93.6%), with 86.4% undergoing vacuum aspiration and 13.6% dilation and curettage. Uterotonic medications were used in 46.8% of MP cases, primarily misoprostol (68.2%) and oxytocin (22.7%) (Table S1).

Over 10% of EP and MP cases were classified as PLTCs or SMO, with EP cases showing a higher prevalence of PLTCs/moderate complications and MP cases more mild complications. The prevalence of PLTCs and SMO among EP and MP cases was higher than that observed in our findings related to abortion‐related complications (4%).^5^ The prevalence of PLTCs/SMO in EP cases in Brazil (~9%) was lower than in Latin America (~14%) and Africa (~30%). MP cases in Brazil had a similar PLTC/SMO prevalence (~6%) to Latin America (~5%) and lower than Africa (~15%).^6^

More than 90% of EP cases required surgery, with laparotomy being the predominant approach. Laparoscopy, a less invasive alternative, has advantages such as reduced blood loss, shorter surgery time, faster recovery, and lower morbidity.^7^ Advances in diagnostic methods like pelvic ultrasound and serum beta‐human chorionic gonadotropin measurement have facilitated early EP diagnosis, potentially preventing tubal rupture and enabling less invasive treatments.^7^ However, in this study, 75% of EP patients had abdominal pain and 34% showed peritoneal irritation at admission, suggesting that many cases were diagnosed late due to limited access to early diagnostic methods. Most MP cases (93%) were managed with uterine evacuation, primarily by manual aspiration (86%), which aligns with treatment guidelines due to its lower risk of heavy bleeding.^8^ About 47% of MP cases also used uterotonic medications, mainly misoprostol, followed by oxytocin.

This study has some limitations. All participating hospitals were public, limiting generalizability to private healthcare settings. MP cases appeared to have fewer complications at discharge, but the absence of follow‐up data prevents assessment of long‐term outcomes like gestational trophoblastic neoplasia. Variations in treatment protocols may have affected management outcomes, particularly regarding medical treatments like methotrexate.

Given the observed prevalence of SMO and PLTCs in EP and MP cases, investments in early diagnostic resources for first‐trimester hemorrhages could reduce these complications in Brazil. Early diagnosis centers could improve timely identification of EP and MP, potentially improving maternal health outcomes.

## AUTHOR CONTRIBUTIONS

Luiz Francisco Baccaro conceptualized the study. Luís Henrique Leão and Camila Ayume Amano Cavalari wrote the first draft. Luís Henrique Leão, Camila Ayume Amano Cavalari, and Luiz Francisco Baccaro reviewed and edited all versions of the manuscript.

## FUNDING INFORMATION

This research was funded by the UNDP‐UNFPA‐UNICEF‐WHO‐World Bank Special Programme of Research, Development and Research Training in Human Reproduction (HRP), Department of Sexual and Reproductive Health and Research, WHO, Geneva, Switzerland. The named authors alone are responsible for the views expressed in this publication and do not necessarily represent the decisions or the policies of the UNDP‐UNFPA‐UNICEF‐WHO‐World Bank Special Programme of Research, Development and Research Training in Human Reproduction (HRP) or WHO, or their individual institutions.

## CONFLICT OF INTEREST STATEMENT

The authors have no conflicts of interest.

## Supporting information


Table S1.

## Data Availability

Research data are not shared.
