# Supplementary material for: Ectopic and molar pregnancies in Brazil: A secondary analysis of the WHO multi‐country survey on abortion
Source: Int J Gynaecol Obstet. 2025 Apr 7;170(3):1423–5. doi: 10.1002/ijgo.70142 (PMC12374019; doi:10.1002/ijgo.70142)
Supplement: Supplementary file 1 — Table S1. [file IJGO-170-1423-s001.docx]

| **Supplemental table 1.** Types of management among women with ectopic and molar pregnancies. | | | | | |
| --- | --- | --- | --- | --- | --- |
| **Types of management** | **Ectopic pregnancy** (%) | **Molar pregnancy** (%) | **Total** | **p-value*** |  |
|  |  |  |  |  |  |
| ***Surgical treatment****^§^* |  |  |  |  |  |
| Uterine evacuation | 1 (0.7) | 44 (93.6) | 45 | <0.01 |  |
| Vacuum aspiration^#^ | 1 (100) | 38 (86.4) | 39 | 1.00 |  |
| Dilation and curettage^#^ | 0 (0.0) | 6 (13.6) | 6 | 1.00 |  |
| Laparotomy | 114 (82.6) | 0 (0.0) | 114 | <0.01 |  |
| Laparoscopy | 6 (4.3) | 0 (0.0) | 6 | 0.34 |  |
|  |  |  |  |  |  |
| ***Clinical treatment****^§^* |  |  |  |  |  |
| Medical treatment^β^ | 35 (25.5) | 14 (29.8) | 49 | 0.57 |  |
| Use of uterotonics | 1 (0.7) | 22 (46.8) | 23 | <0.01 |  |
| Use of IV fluids | 129 (94.2) | 34 (72.3) | 163 | <0.01 |  |
| Use of vasopressors | 3 (2.2) | 0 (0.0) | 3 | 0.57 |  |
| Use of antibiotics | 83 (60.6) | 20 (42.5) | 184 | 0.03 |  |
| Procoagulant agents | 5 (3.6) | 1 (2.1) | 6 | 1.00 |  |
| Blood transfusion | 16 (11.7) | 3 (6.4) | 19 | 0.41 |  |
| ICU admission | 2 (1.45) | 0 (0.0) | 2 | 1.00 |  |
| Prolonged stay | 45 (33.1) | 6 (12.8) | 51 | <0.01 |  |

*Chi-square test for comparison between ectopic pregnancy and molar pregnancy.

^β^Includes methotrexate or another similar form for molar or ectopic pregnancies

^§^Because women could receive more than one surgical or clinical treatment, totals do not add up

^#^Among women submitted to uterine evacuation
